# Comprehensive Analysis of Treatment Approaches in Chest Wall Ewing Sarcoma: The Impact of Tumor Volume on Oncologic Outcomes

**DOI:** 10.1016/j.adro.2025.101729

**Published:** 2025-02-28

**Authors:** Brady S. Laughlin, Aaron Bogan, Wendy A. Allen-Rhoades, Peter S. Rose, Stephanie F. Polites, Jonathan B. Ashman, Ivy Petersen, Michael G. Haddock, Anita Mahajan, Nadia N. Laack, Safia K. Ahmed

**Affiliations:** a Department of Radiation Oncology, Mayo Clinic, Phoenix, Arizona; b Department of Quantitative Health Sciences, Mayo Clinic, Scottsdale, Arizona; c Department of Pediatric Hematology/Oncology, Mayo Clinic, Rochester, Minnesota; d Department of Orthopedic Surgery, Mayo Clinic, Rochester, Minnesota; e Department of Pediatric Surgery, Mayo Clinic, Rochester, Minnesota; f Department of Radiation Oncology, Mayo Clinic, Rochester, Minnesota

## Abstract

**Purpose:**

Local treatment with surgery (S) and radiation therapy (RT) for chest wall Ewing sarcoma (cwES) is often challenging given the extent of the tumor and the aggressiveness of local treatments needed for cure. We report tumor and treatment characteristics, oncologic outcomes, and toxicities of patients with cwES at 2 centers of a single institution.

**Methods and Materials:**

Consecutive patients with cwES treated from 1997 to 2022 were retrospectively reviewed. All patients were treated with standard 5-drug chemotherapy (vincristine, doxorubicin, cyclophosphamide, alternating with ifosfamide and etoposide) before initiation of local therapy. Local treatment was S, RT, or both. The decision on modality and timing was determined by a multidisciplinary sarcoma group or by consensus between sarcoma experts regarding patient preferences.

**Results:**

The cohort consisted of 39 patients. The median age at diagnosis was 19.2 years (range, 3.5-53.6 years). Median tumor volume (TV) was 235.5 mL (range, 5.3-6761.9 mL). The local control (LC) modality was S in 18 patients (46%), RT in 4 (10%), and S + RT in 17 (44%). Four (10%) patients treated with S + RT had R1 margins. The median follow-up was 3.2 years (range, 0.1-21.6 years). Grade 3 radiation-associated toxicity relative to the RT modality was 16.7% and 7.1% for photons (n = 6) and protons (n = 14), respectively. The 2-year LC by modality was 100% for RT (95% CI, 100%-100%), 88.2% (95% CI, 74.2%-100%) for S, and 73.3% (95% CI, 54.0%-99.5%) for S + RT. The 5-year LC, failure-free survival, and overall survival for all patients were 79.7% (95% CI, 67.3%-94.4%), 52.3% (95% CI, 38.1%-71.9%), and 64.2% (95% CI, 49.6%-83.1%), respectively. In univariate and multivariate analysis, TV ≥ 130 mL was associated with a significantly worse 5-year failure-free survival (31.8% TV ≥ 130 mL vs 80.8% TV < 130 mL; hazard ratio, 4.94, *p* = .013 and adjusted hazard ratio, 5.43; 95% CI, 1.28-22.98; *p* = .022). The multivariate model was adjusted for age, metastatic disease at diagnosis, and S.

**Conclusions:**

Outcomes for cwES tumors are highly dependent on tumor size, even with the use of combined modality local therapy. With early follow-up, smaller tumors may be well controlled with either S or RT.

## Introduction

Chest wall Ewing sarcoma (cwES) is a rare entity, accounting for approximately 13% to 20% of all Ewing sarcoma cases. Given the rare occurrence of these tumors, the optimal local treatment approach needs to be better established. Local control (LC) strategies are often individualized and depend on the extent and location of the disease. When patients have metastatic disease involving unilateral or bilateral lungs, hemithorax or whole lung irradiation is required in addition to focal site treatment. Outcome data indicate that despite aggressive treatment, there is a high propensity for local and distant relapse, highlighting the need for improved therapeutic strategies.^1^, ^2^, ^3^, ^4^

Given the propensity for relapse, intensive therapy is required to control for both local and distant relapse.^3^ Local treatment approaches for cwES are challenging, given that these tumors frequently present as large chest wall tumors and that they often grow into the thoracic cavity before patients become symptomatic. The extent of local disease can also include pleural effusions, pleural nodules, lymphadenopathy, and pulmonary metastases. As such, local treatment with both surgery (S) and radiation therapy (RT) can be challenging given the extent of the tumor, the presence of adjacent critical organs, and the aggressiveness of local treatments (eg, treatment of large fields for RT and extensive resections to obtain a negative margin with S) needed for a cure. Furthermore, local treatment is associated with high-toxicity rates.^1^^,^^4^

Our study aimed to contribute to the limited cwES literature by evaluating treatment characteristics and oncologic outcomes in a cohort of patients treated by a collaborative sarcoma group. We report a 25-year retrospective study to evaluate the efficacy of S, RT, and S + RT for local tumor control. We also assessed survival outcomes, treatment-associated toxicities, and factors associated with prognosis.

## Methods and Materials

### Patient cohort

A retrospective chart review (approved by the institutional review board, number 09-008050) of consecutive cwES cases with local treatment between 1997 and 2022 performed at 2 centers of a single institution was completed. Chest wall tumors were defined as tumors involving the ribs, sternum, or pleura. Pertinent patient, tumor, treatment, toxicity, survival, and relapse data were extracted from the medical record. Tumor diameter and volume at diagnosis were recorded. The maximum tumor dimension was recorded as less than or greater than 8 cm. The tumor volume (TV) was calculated using the formula used in the Children's Oncology Group (COG) AEWS1031 trial: 0.5* (d1 * d2 * d3) and was characterized as less than, greater than, or equal to 130 mL at the time of diagnosis.

Data regarding dose-dense and interval-compressed chemotherapy were unavailable as many patients had received chemotherapy at outside institutions. The decision on modality and timing was determined by a multidisciplinary sarcoma group or by consensus between sarcoma experts regarding patient preferences. In patients who underwent surgical resection, the goal was to resect the tumor with grossly negative margins. Microscopic negative margins were defined as R0 and microscopic positive margins as R1. Patients who underwent an R1 resection were recommended to have postoperative RT. Definitive RT was recommended in patients not treated with S. Preoperative radiation was considered in patients with poor response to chemotherapy as well as in patients with an anticipated close-margin resection. These patients included patients where an R0 or R1 resection was deemed to be unattainable or in patients where S would cause significant morbidity. Postoperative RT was considered in patients who have an R1 resection or close margins. The decision regarding postoperative RT was made at the discretion of the treating radiation oncologist in consultation with the multidisciplinary team.

Patients treated with RT were classified as receiving proton beam therapy (PBT) or photon therapy, either 3-dimensional conformal therapy or intensity modulated RT. The choice of radiation modality was at the physician's discretion. The use of PBT for primary site radiation was prioritized to help meet lung dosimetric constraints.

During the simulation, patients were immobilized with a 5-point thermoplastic mask covering the head and shoulders while keeping the arms down.^5^ A 4-dimensional computed tomography (CT) was performed to assess for respiratory motion. When diaphragmatic motion exceeded 1.0 cm, active motion management was implemented using either breath-hold or combined phase-gating with layer-based rescanning.^5^ For patients with motion <1.0 cm, only the rescanning technique was employed.^5^ Contouring was completed on the average scan for patients with minimal motion, whereas for those with significant respiratory motion, target delineation was performed on the phase subset average scan corresponding to the gated phases or on the breath-hold scan if applicable.^5^

Treatment volumes followed COG guidelines. The gross TV (GTV), clinical target volume (CTV), internal target volume, and planning target volumes (PTV) were created. GTV1 was defined as a visible or palpable disease on physical examination, CT, magnetic resonance imaging, or positron emission therapy scan before S or chemotherapy. GTV1 was modified if it exhibited a pushing margin into the thorax. If the tumor responded to chemotherapy, GTV1 excluded the prechemotherapy volume, which extended into the cavity. CTV1 was defined as the GTV1 plus 1 cm margin. CTV1 also included regional lymph node chains for clinically or pathologically involved nodes. GTV2 was defined as a residual tumor as assessed by CT, magnetic resonance imaging, positron emission therapy scan, or physical examination following induction chemotherapy with or without S. GTV included the pretreatment disease extent in bone for unresected tumors and gross residual extent in soft tissue following induction chemotherapy. CTV2 was defined as GTV2 + 1 cm and areas at risk for microscopic disease. CTV2 was modified to account for specific anatomic barriers to tumor spread. Target motion was accounted for with all treatment volumes. If a patient received whole lung irradiation or hemithorax radiation, the target volumes were designated as CTV3 and PTV3.

Normal organ-at-risk dose recommendations were followed and prioritized for minimization of toxicity. For patients not receiving hemithorax or whole lung RT, a bilateral lung V20 Gy (volume receiving 20 Gy) < 20% was prioritized. For patients requiring hemithorax or whole lung RT and boost treatment of the chest wall or pleura, a bilateral V20 Gy of 35% was prioritized.

Treatment-related toxicity was evaluated through a review of follow-up visits and categorized per the Common Terminology Criteria for Adverse Events version 5.0. Time to failure endpoints for overall survival (OS) were calculated from the date of S or the end of RT to the date of the last follow-up or death. LC was defined as the time from the end of the LC modality (S or RT) to local relapse. Distant metastatic disease was defined as the date of local modality to distant relapse. In patients with pulmonary metastases, developing additional pulmonary metastases in the RT field was considered a local failure (LF). Similarly, patients with pleural nodules or pleural effusion who developed subsequent pleural or pleural effusion were counted as having LF. Failure-free survival (FFS) was defined as the time from the end of the LC modality to local or distant relapse or death.

### Statistical analysis

Kaplan-Meier and Cox proportional hazard models were used to evaluate factors associated with time-to-event occurrence (OS, LC, and FFS). *P* values from univariate and multivariate Cox regressions and Kaplan-Meier log-rank tests were used to identify variables significantly associated with OS, LC, and FFS. Kaplan-Meier hazard ratios (HRs) and 95% CIs were used to determine the event risk (OS, LC, and FFS) as a function of time. Analyses were performed using R version 4.2.2.^5^ *P* values were derived from 2-tailed tests and were considered statistically significant if they were <.05.

## Results

Thirty-nine patients were identified from the review of medical records and the cancer center registry. Table 1 reports patient, tumor, and treatment characteristics. The median age at diagnosis was 19.2 years (range, 3.5-53.6 years; IQR, 12.9 years). The most common tumor sites were ribs 8 to 12 (64.1%) and ribs 4 to 7 (23.1%). A total of 13 (33.3%) patients presented with metastatic disease: 5 (12.8%) patients presented with pleural effusion, 5 (12.8%) patients presented with pleural nodules, 6 (15.4%) patients with lung metastases, and 1 (2.6%) patient had extrathoracic metastasis. The median TV was 235.5 mL (range, 5.3-6761.9 mL; IQR, 298.8 mL), and 21 (56.8%) tumors were ≥ 130 cm3. All patients (100%) were treated with standard 5-drug chemotherapy (vincristine, doxorubicin, cyclophosphamide, alternating with ifosfamide and etoposide [VDC/IE]).

**Table 1:** Patient and treatment characteristics

	RT alone (n = 4) n (%)*	Surgery + RT (n = 17) n (%)*	Surgery alone (n = 18) n (%)*	Total (N = 39) n (%)*	*P* value
Patient characteristics					
Sex					
Male	2 (50.0)	9 (52.9)	8 (44.4)	19 (48.7)	-
Female	2 (50.0)	8 (47.1)	10 (55.6)	20 (51.3)	-
Age at diagnosis	.382†				
Mean (SD)	20.5 (5.5)	18.7 (10.6)	24.6 (14.0)	21.6 (12.1)	-
Median	21.9	16.5	20.7	19.2	-
IQR	5.0	9.9	18.7	12.9	-
Range	12.7-25.4	7.5-50.0	3.5-53.6	3.5-53.6	-
Location of local disease					
Ribs 1-3	0 (0.0)	1 (5.9)	1 (5.6)	2 (5.1)	-
Ribs 4-7	3 (75.0)	2 (11.8)	4 (22.2)	9 (23.1)	-
Ribs 8-12	1 (25.0)	12 (70.6)	12 (66.7)	25 (64.1)	-
Pleura	0 (0.0)	2 (11.8)	1 (5.6)	3 (7.7)	-
Any metastasis					
No	3 (75.0)	8 (47.1)	15 (83.3)	26 (66.7)	-
Yes	1 (25.0)	9 (52.9)	3 (16.7)	13 (33.3)	-
Tumor volume (cc)	.194†				
Mean (SD)	217.8 (178.3)	1157.9 (2004.2)	199.9 (257.3)	641.5 (1431.6)	-
Median	266.3	290.9	85.9	235.5	-
IQR	173.3	447.7	217.3	298.8	-
Range	20.4-366.9	5.3-6761.9	12.4-1037.8	5.3-6761.9	-
Missing	1	0	1	2	-
Tumor volume (cc), grouped					
<130	1 (33.3)	4 (23.5)	11 (64.7)	16 (43.2)	-
≥130	2 (66.7)	13 (76.5)	6 (35.3)	21 (56.8)	-
Missing	1	0	1	2	-
Maximum tumor diameter (cm)	.105†				
Mean (SD)	8.8 (3.0)	12.9 (9.1)	7.8 (2.5)	10.2 (6.8)	-
Median	10.0	9.6	6.9	8.7	-
IQR	2.8	3.6	3.5	4.3	-
Range	5.4-11.0	2.9-33.8	4.0-13.9	2.9-33.8	-
Missing	1	0	1	2	-
Maximum tumor diameter (cm), grouped					
<8	1 (33.3)	4 (23.5)	10 (58.8)	15 (40.5)	-
≥8	2 (66.7)	13 (76.5)	7 (41.2)	22 (59.5)	-
Missing	1	0	1	2	-
Metastatic disease outside thorax					
No	4 (100.0)	17 (100.0)	16 (88.9)	37 (94.9)	-
Yes	0 (0.0)	0 (0.0)	2 (11.1)	2 (5.1)	-
*Radiationdetails*					
Radiation modality					
Photon	1 (25.0)	5 (29.4)		6 (28.6)	-
Proton	3 (75.0)	11 (64.7)		14 (66.7)	-
Both	0 (0.0)	1 (5.9)		1 (4.8)	-
Missing	0	0		18	-
Radiation timing					
Preoperative	0 (0.0)	3 (17.6)		3 (14.3)	-
Definitive	4 (100.0)	0 (0.0)		4 (19.0)	-
Postoperative	0 (0.0)	14 (82.4)		14 (66.7)	-
Missing	0	0		18	-

*Abbreviation:* RT = radiation therapy.

⁎ Unless otherwise specified.

† Kruskal-Wallis rank sum test.

The LC modality was S in 18 patients (46.2%), RT in 4 (10.3%), and S + RT in 17 (43.6%). The median TV was 85.9 mL (range, 12.4-1037.8 mL; IQR, 217.3 mL) for S patients, 266.3 mL (range, 20.4-366.9 mL; IQR, 173.3 mL) for RT patients, and 290.9 mL (range, 5.3-6761.9 mL; IQR, 447.7 mL) for S + RT patients (*p* = .19). S primarily consisted of en bloc chest wall tumor resection with the inclusion of associated soft tissue and portions of the lung or diaphragm if deemed necessary. Of the patients undergoing S, 23 (66%) underwent rib resections. The median number of ribs resected was 3 (range, 1-5). There were 9 (26%) and 3 (9%) patients who had lung and diaphragmatic resections, respectively. There was 1 (3%) patient with pleural nodules resected. There were 8 (20%) patients who underwent chest wall reconstruction. R0 and R1 resections were performed in 31 (88.6%) and 4 (11.4%) of the S patients, respectively. There were no surgical patients with macroscopic positive margins.

**Figure 1:**
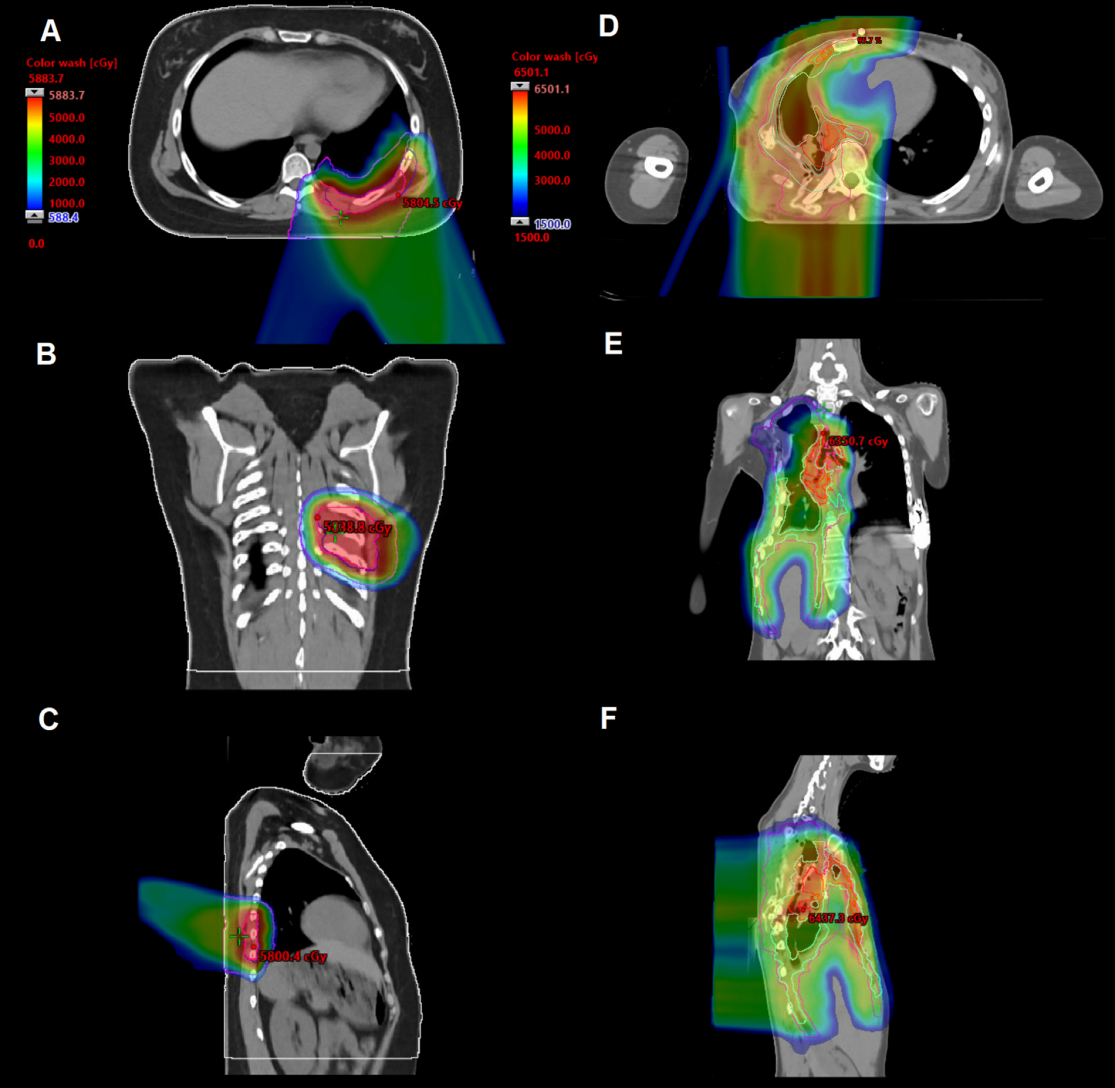
Case examples of localized (A-C) and hemithorax radiation (D-F) with proton beam therapy. The first patient received definitive radiation of 55.8 Gy in 31 fractions to clinical target volume (CTV) 1 and 45 Gy in 25 fractions to CTV2. The second patient received 54.6 Gy to CTV1, 49.6 Gy to CTV2, and 15 Gy to CTV3 (right hemithorax).

For patients undergoing RT, radiation was delivered definitively in 4 (19.0%) patients, postoperatively in 14 (66.7%) patients, and preoperatively in 3 (14.3%) patients. In patients undergoing definitive radiation, PBT was delivered in 3 (75%) patients and photons in 1 (25%) patient. Nine (64.3%) patients who received radiation postoperatively were delivered protons, 4 (28.6%) were delivered photons, and 1 (7.1%) was delivered a combination of both. PBT was used in 14 (66.7%) patients. For patients receiving RT, the median dose to GTV1/CTV1/PTV1 was 45 Gy (range, 4000-5000 cGy). The median dose was 53.2 Gy (range, 45-60 Gy; IQR, 5.4 Gy) in 29 fractions to GTV2/CTV2/PTV2. The median dose to CTV3/PTV3 for hemithorax (4 patients, 19.0%) (Fig. 1D-F) and whole lung irradiation (4 patients, 19.0%) was 15.0 Gy (range, 12.0-18.0 Gy) in 10 fractions. Hemithorax radiation was protons in 3 (14.3%) of the 21 RT patients. One patient received photons for hemithorax RT followed by an involved field boost with protons (4.8% of RT patients). Whole lung radiation was protons in 3 (14.3%) patients and photons in 1 (4.8%) patient. Three (14.3%) RT patients were treated with preoperative radiation before resection (range, 45-55.8 Gy). A patient with a very poor response to neoadjuvant chemotherapy was treated with 36 Gy in 20 fractions preoperatively and then a 20 Gy boost to the postoperative bed for a microscopic positive margin. For all patients receiving RT, the median bilateral lung V20 Gy was 12.9% (range, 1.0%-35.3%; IQR, 21.6%). For patients receiving localized RT, the median lung V20 Gy was 12.9% (range, 1.0%-35.3%; IQR, 21.6%). For patients receiving hemithorax/whole lung RT, the median lung V20 Gy equaled 26.9% (range, 1.7%-35.3%; IQR, 8.7%).

**Figure 2:**
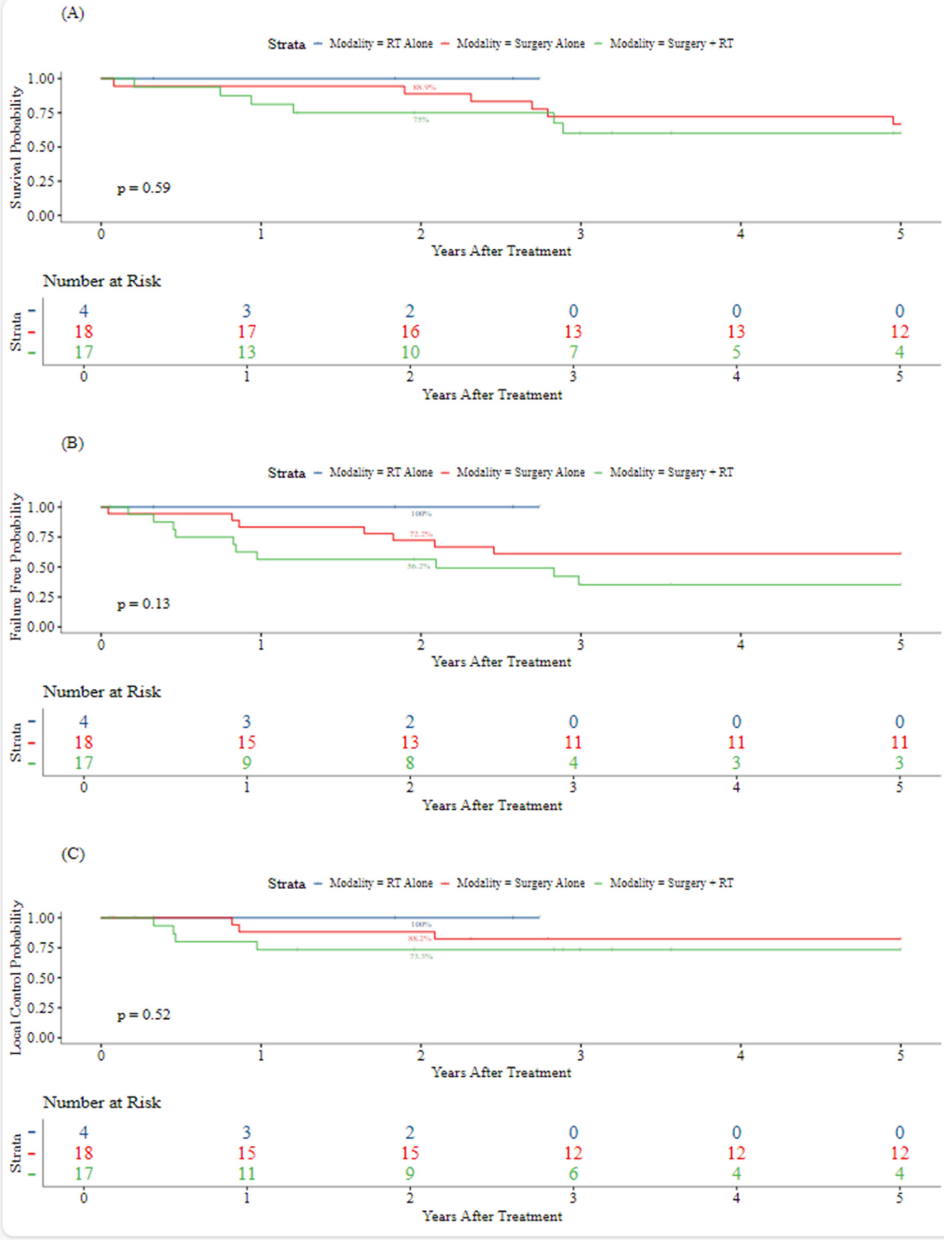
Kaplan-Meier curves for 5-year overall survival (A), failure-free survival (B), and local control (C) by modality. *Abbreviations:* RT = radiation therapy.

**Figure 3:**
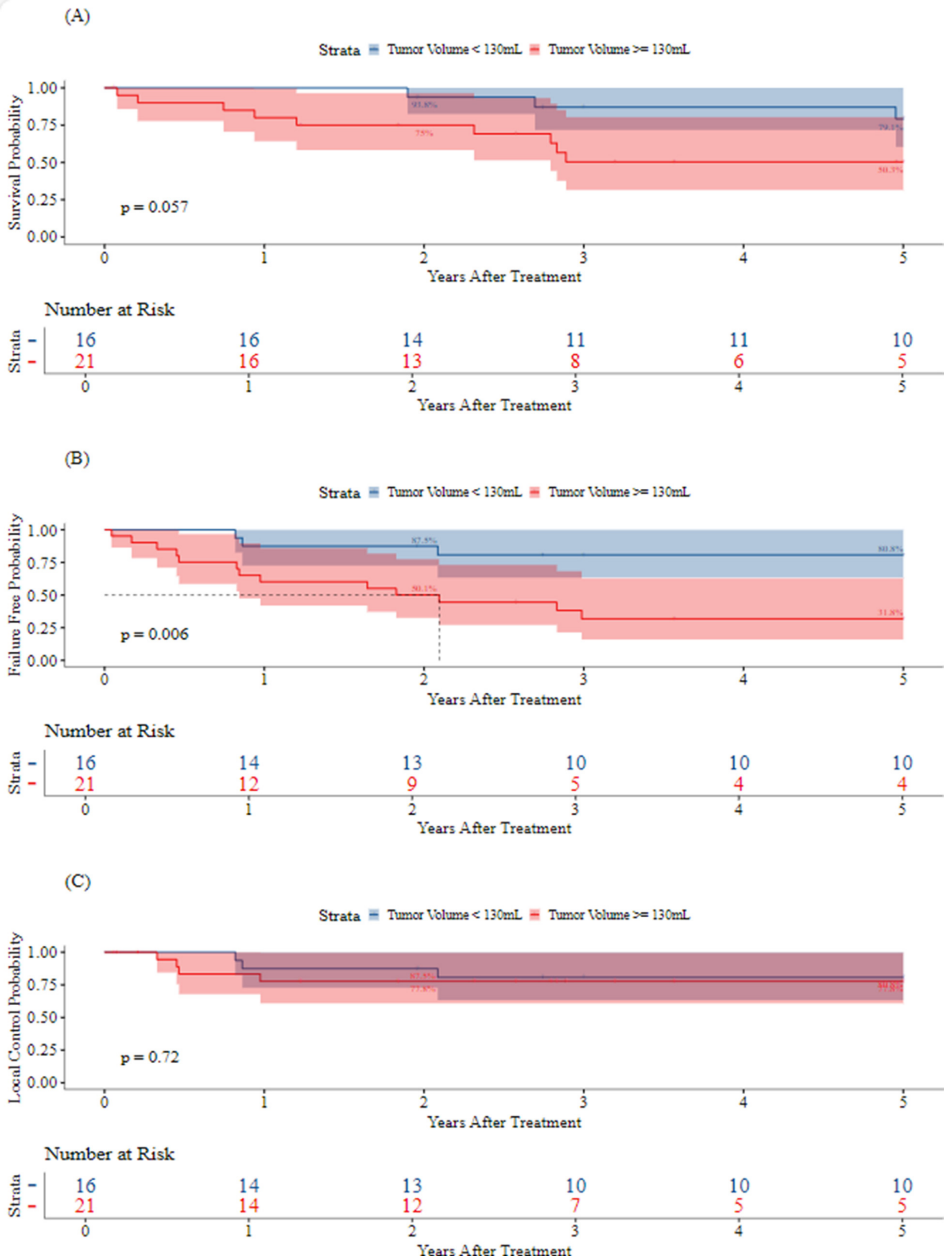
Kaplan-Meier curves for 5-year overall survival (A), failure-free survival (B), and local control (C) by tumor volume.

**Figure 4:**
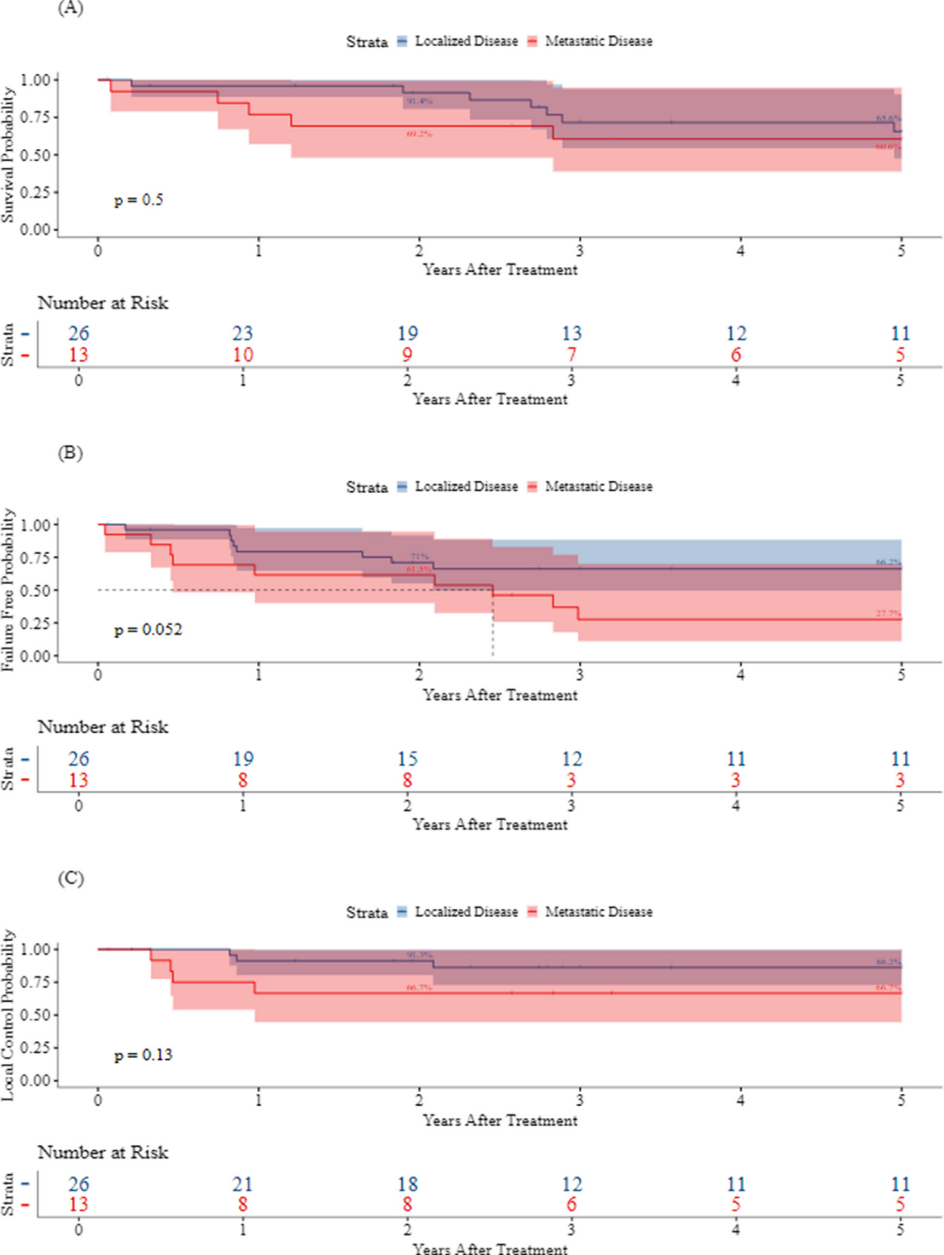
Kaplan-Meier curves for overall survival (A), failure-free survival (B), and local control (C) by metastatic disease status.

The median follow-up was 3.2 years (range, 0.1-21.6 years; IQR, 6.8 years). The five-year LC, FFS, and OS for all patients were 79.7% (95% CI, 67.3%-94.4%), 52.3% (95% CI, 38.1%-71.9%), and 64.2% (95% CI, 49.6%-83.1%), respectively. Figure 2, Figure 3 demonstrate Kaplan-Meier curves for LC, FFS, and OS by LC modality and TV, respectively. As the first event, LF occurred in 5 patients (1, S; 4, S + RT). There were no LFs in patients who received RT alone, including those who received whole lung irradiation or hemithorax RT. Two-year LC by modality was 100% for RT (95% CI, 100%-100%), 88.2% (95% CI, 74.2%-100%) for S, and 73.3% (95% CI, 54%-99.5%) for S + RT. Metastatic disease developed in 13 (33.3%) patients. Metastasis was the first event in 10 (25.6%) patients. There were 3 (7.7%) patients who developed LF and metastatic disease. Seven (17.9%) and 6 (42.1%) patients who developed metastatic disease received S alone and S + RT, respectively. The 5-year rates of LC, FFS, and OS were 86.2% (95% CI, 72.8%-100%), 66.2% (95% CI, 49.6%-88.4%), and 65.6% (95% CI, 47.6%-90.4%), respectively, in patients with localized disease, and 66.7% (95% CI, 44.7%-99.5%), 27.7% (95% CI, 11%-69.9%), and 60.6% (95% CI, 38.7%-94.7%), respectively, in patients with metastatic disease at baseline (Fig. 4).

**Figure 5:**
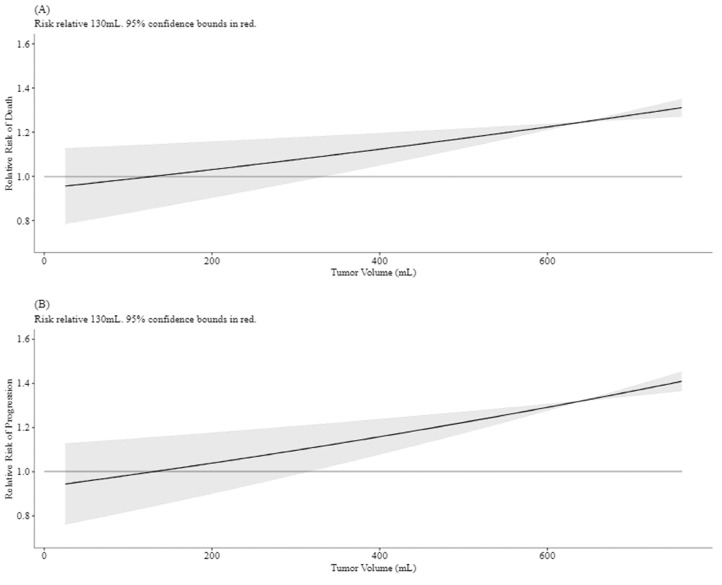
Relative risk of overall survival (A) and failure-free survival (B) by tumor volume.

Figure 5 demonstrates the relative risk of progression and OS as a function of TV at diagnosis. In univariate analysis, TV ≥ 130 mL was associated with a significantly worse 5-year FFS (31.8% TV ≥ 130 mL vs 80.8% TV < 130 mL; HR, 4.94; *p* = .013). TV ≥ 130 mL was not statistically significantly associated with OS at the .05 level (HR, 3.33; 95% CI, 0.90-12.38; *p* = .072). Maximum tumor diameter ≥ 8 cm was associated with significantly worse FFS (HR, 4.57; 95% CI, 1.29-16.17; *p* = .018) as well as OS (HR, 5.08; 95% CI, 1.11-23.33; *p* = .036). Metastatic disease at diagnosis, positive surgical margin, PBT, and LC modality did not significantly impact OS, FFS, or LC. In multivariate analysis, TV ≥ 130 mL remained a significant predictor of worse FFS when adjusting for age at diagnosis, metastatic disease at diagnosis, and S (adjusted HR, 5.43; 95% CI, 1.28-22.98; *p* = .022).

There was no significant difference in treatment toxicity by modality (PBT vs photon). For the 35 surgical patients, surgical toxicity included 1 (2.9%) grade 3 chyle leak, 3 (8.6%) grade 3 infections, 2 (5.7%) grade 3 wound complications, and 1 (2.2%) grade 3 air leak. For the 21 patients receiving RT, there were low rates of acute grade 2+ toxicity following the completion of RT: 2 (9.5%) grade 2 and 1 grade 3 (4.8%) fatigue and 2 (9.5%) grade 2 radiation dermatitis. Grade 3 radiation-associated toxicity relative to RT modality was 16.7% (1 grade 3 fatigue) and 7.1% (1 grade 3 pneumonia) for photons (n = 6) and protons (n = 14), respectively. Late grade 2+ toxicity consisted of 1 (2.6%) patient with chronic kidney disease because of chemotherapy, 4 (10.3%) patients with scoliosis, and 1 (2.6%) with chest wall deformity. There were no patients who developed pneumonitis.

## Discussion

CwES tumors have historically been associated with poor outcomes, given the extensive tumor burden often seen with these tumors and the suboptimal systemic therapy and challenges with local therapies. CwES from the literature reports a wide range of 5-year LC, event-free survival (EFS), and OS from 72% to 97%, 34% to 74%, and 34% to 81.6%, respectively.^1^, ^2^, ^3^^,^^6^^,^^7^ Our manuscript contributes to the current literature by describing local therapy approaches and outcomes in a large cohort of cwES. The use of PBT needs further exploration, potentially allowing for dose escalation without increasing toxicity.

Our results suggest that larger tumors are associated with worse oncologic outcomes regardless of the local therapy modality employed. Large tumor size is a known critical prognostic factor that has been shown to correlate with LC.^8^^,^^9^ The COG LF analysis of patients treated in INT-0091, INT-0154, and AEWS0031 showed that tumor size was not associated with local recurrence.^10^ However, it is noteworthy that tumor size was not recorded for 60% of the patient cohort, impacting the robustness of these findings.^10^ In AEWS1031, where vincristine, topotecan, and cyclophosphamide were added to standard VDC/IE for nonmetastatic Ewing sarcoma, TV > 200 mL was associated with a significantly lower EFS.^11^ In addition to large tumor size, several prognostic factors were identified in both North America and Europe, including axial sites with pelvic tumors,^9^^,^^12^^,^^13^ age > 13 years old,^9^^,^^12^^,^^14^ male sex,^15^ and poor response to neoadjuvant chemotherapy.^16^, ^17^, ^18^ Our data correlate to an extent, notably that large TVs (>130 cm3) are associated with worse FFS. However, we could not perform multivariate analysis for metastatic disease because of our limited sample size and the interaction of various factors. We acknowledge that metastatic disease is the most important prognostic factor for Ewing's sarcoma (ES), and our results suggest a difference in 5-year FFS for patients with localized versus metastatic disease of 66.2% (95% CI, 49.6%-88.4%) versus 27.7% (95% CI, 11.0%-69.9%). Given our limited sample size, age did not predict outcomes in patients with cwES.

Our findings suggest that individualized LC strategies tailored to the extent of the disease play a critical role in the treatment of cwES and impact FFS. Our study is not the first to compare S alone, RT alone, and S + RT for managing cwES. A retrospective analysis of the Cooperative Ewing’s Sarcoma Studies CESS 81, CESS 86, and the European Intergroup Ewing’s Sarcoma Study EICESS 92 provides the most extensive series of patients with cwES. With 114 patients, S was performed in 14 patients, RT alone in 28 patients, and S + RT in 71 patients. For the entire cohort, the OS was 60%, and EFS was 50%.^19^ In this series, LF occurred in 0% after S alone, 19% after RT alone, and 19% after postoperative irradiation. Eight patients received preoperative radiation, none of which developed LF.^19^ Our series compares similarly with a 5-year OS of 62.1%. However, our series compares similarly to those patients receiving S alone. In patients who received S alone as an LC modality, we report a 12% and 18% LF rate at 2 years and 5 years, respectively, compared with a 7.5% rate from the combination of the CESS 81, CESS 86, and EICESS 92 studies.^19^ As primary local treatment is deferred until after induction chemotherapy, higher rates of complete resection may be obtained, reducing the need for adjuvant RT. In a retrospective study of 89 patients with primary cwES or primitive neuroectodermal tumor from INT0091 and POG9354, rates of 5-year EFS were roughly 65%, 55%, and 30% for S alone, S + RT, and RT alone, respectively. As patients usually receiving adjuvant radiation often consist of close or positive margins, large tumors, or poor response to chemotherapy, it is not surprising that patients receiving S + RT had worse outcomes overall in our study.

Traditionally, historical data have demonstrated that S improves outcomes in select patients compared with definitive RT.^20^^,^^21^ In a combined analysis of INT0091, INT0154, and AEWS0031 from the COG, patients undergoing S tend to have favorable characteristics such as younger age and extremity site.^21^ Additionally, radiation was typically used more frequently in earlier decades with older techniques.^21^ In a comparative evaluation of LC modalities for Ewing sarcoma, definitive radiation was associated with a significantly inferior EFS (HR, 1.7; *P* = .004) compared with S. However, the difference was no longer significant for EFS in multivariate analysis. On the other hand, LC may vary depending on modality, tumor site, and other factors.^21^ Per the COG analysis of INT0091, INT0154, and AEWS0031, S had higher LC rates than radiation alone for extremity and pelvic tumors (3.7% and 3.9% vs 14.8% and 22.4%, respectively).^22^ There was no significant difference in LC observed after S for the spine or other axial nonspine, including chest wall tumors and extraskeletal tumors.^22^ Our study is comparable with the literature in that S alone fared similarly to surgical outcomes reported in the literature. This is likely because S alone is performed for small chest wall tumors. Larger chest wall tumors require complex surgical resection, with a higher risk of residual microscopic disease, contributing to our study's higher rates of local recurrence and overall failure in the S + RT group.

CwES are often only present after growing large enough to cause symptoms such as respiratory distress or pain. Additionally, disease burden may include pleural effusions, pleural nodules, lymphadenopathy, and pulmonary metastases. As a result, RT employs extensive fields and varying doses using a dose painting technique to target the extent of the disease effectively. If treated definitively, the prechemotherapy extent of the disease is treated to 45 Gy in 25 fractions, followed by a boost to prechemotherapy bone involvement and postchemotherapy soft tissue extent to 55.8 Gy over 31 fractions. Postoperatively, the primary site is treated to 50.4 Gy if positive or close margins exist. The European approach determines the dose depending on margin status and histologic response, ranging between 45 and 54 Gy. If there are pulmonary metastases, the involved lung is treated to 15 Gy in 10 fractions. Involved lymph nodes and pleural nodules traditionally receive 45 Gy in 25 fractions. Because large chest wall tumors may require extensive radiation fields, PBT is advantageous because of its ability to minimize exposure to healthy tissue. Our study demonstrated low rates of high-grade toxicity and no evidence of grade 2 or higher pneumonitis in our patient cohort.

In AEWS1221, patients with metastatic Ewing's sarcoma were randomized to standard interval-compressed VDC/IE with or without ganitumab. The incidence of grade 3+ pneumonitis occurred in 0.7% and 2.7% of patients in the standard and ganitumab arms, respectively. However, more cases of pneumonitis occurred in patients treated with thoracic fields.^23^ To mitigate the risk of pneumonitis, lung parameters must be strictly followed; using PBT has a distinct advantage in this situation. AEWS1221 recommends a bilateral lung V20 Gy < 20% for patients not receiving hemithorax RT. A bilateral V20 Gy of 35% was followed for patients receiving hemithorax RT. In our study, we did not see any evidence of grade 2+ pneumonitis in our patient cohort. This may be attributable to the strict adherence to the planning parameters and the use of PBT in most patients receiving radiation. Additionally, gross tumor resection before radiation can also assist with minimizing the volume of lung tissue exposed to the higher boost radiation doses, as 42% of our series was treated with S + RT. Incorporating PBT into multimodality treatment for cwES offers the potential to improve LC and reduce treatment-related toxicity.

As therapy has improved over time, we have seen improvements in oncologic outcomes for cwES. Indelicato et al^1^ initially reported on 39 patients (22 RT alone and 17 S + RT) between 1966 and 2009. For all patients, the 5-year LC, cause-specific survival, and OS were 72%, 34%, and 34%, respectively.^1^ A difference was noted when evaluating LC between S + RT (75%) and RT alone (61%), although not statistically significant. While aggressive management is needed to demonstrate these outcomes, it comes at the expense of higher toxicity. Indelicato et al^1^ reported a high rate of grade 3+ toxicity of 26%. Eight complications were attributable to RT, delivered with photon techniques. Additionally, 3 of 7 deaths occurred in patients receiving total or hemithorax RT. In their series from the modern era, Indelicato et al^2^ reported outcomes of patients receiving PBT for cwES, not including patients treated with S alone. They report significantly higher 5-year outcomes in 39 patients. With a median follow-up of 4 years, the 5-year LC, FFS, and OS were 97.2%, 74.4%, and 81.6%, respectively.^2^ In addition to favorable outcomes for this patient population, Indelicato et al^2^ reported low rates of high-grade toxicity: 2 patients developed grade 2 pneumonitis, and 2 developed grade 3 scoliosis requiring corrective S. Our series spans 25 years and includes patients in the modern era. Therefore, the disease outcomes of our patient cohort fall in between those reported in the studies by Indelicato et al.^2^ Overall, the entire cohort's 5-year LC, FFS, and OS were 79.7%, 52.3%, and 64.2%, respectively. Additionally, our study differs in that it includes patients treated with S alone. We do not have extended follow-ups in patients treated solely with PBT (RT alone), but we see 100% LC at 2 years. Toxicity was also limited, and no patients developed pneumonitis.

Past studies have demonstrated that tumor size or volume at diagnosis, specific subsites, and response to neoadjuvant chemotherapy are associated with worse progression-free survival and OS.^8^^,^^10^^,^^9^^,^^19^ In AEWS1031, TV > 200 mL was significantly associated with worse EFS.^11^ A strength of our study is the demonstrated association between cwES tumor size and worse FFS. Although not statistically significant, cwES >200 mL demonstrated worse OS and LC. Patients undergoing S + RT demonstrated worse clinical outcomes, more likely as a result that these were larger tumors. Larger chest wall tumors were more likely to be treated with a combined approach of S + RT or RT dose escalation.^24^^,^^25^ In our study, patients receiving S + RT had a clinically meaningful difference in TV compared with S or RT alone, despite not being statistically significant (*p* = .09). However, most proceed with extreme caution with RT dose escalation, given the risk of pneumonitis. PBT should be pursued for RT dose escalation cases.

This retrospective study includes limitations. This study includes a small sample size in which various local modalities were used: S alone, RT alone, and S + RT. Additionally, as time has progressed, there have been advancements in RT techniques, such as PBT, which allows for more tissue sparing. Although this is a retrospective study spanning 25 years, the cohort has a short median follow-up time. Unfortunately, multiple patients were lost to follow-up, and many patients resumed follow-up following completion of therapy closer to home. Therefore, oncologic outcome analysis by LC modality can only be performed at the 2-year timepoint. Future studies will need more patients with more extended follow-up periods.

## Conclusions

cwES with TV greater than or equal to 130 mL had worse FFS and clinically significantly worse OS and inferior LC. The findings of this study demonstrate the prognostic significance of tumor size, with larger tumors leading to inferior outcomes. These results emphasize the challenges of cwES tumors and underscore the role of a multidisciplinary approach to optimize outcomes while minimizing treatment-related toxicity.

## Disclosures

The authors declare that they have no known competing financial interests or personal relationships that could have appeared to influence the work reported in this paper.
